# Interacting Social and Environmental Predictors for the Spatial Distribution of Conservation Lands

**DOI:** 10.1371/journal.pone.0140540

**Published:** 2015-10-14

**Authors:** Robert F. Baldwin, Paul B. Leonard

**Affiliations:** Department of Forestry and Environmental Conservation, Clemson University, 261 Lehotsky Hall, Clemson, SC 29634, United States of America; University of Waterloo, CANADA

## Abstract

Conservation decisions should be evaluated for how they meet conservation goals at multiple spatial extents. Conservation easements are land use decisions resulting from a combination of social and environmental conditions. An emerging area of research is the evaluation of spatial distribution of easements and their spatial correlates. We tested the relative influence of interacting social and environmental variables on the spatial distribution of conservation easements by ownership category and conservation status. For the Appalachian region of the United States, an area with a long history of human occupation and complex land uses including public-private conservation, we found that settlement, economic, topographic, and environmental data associated with spatial distribution of easements (N = 4813). Compared to random locations, easements were more likely to be found in lower elevations, in areas of greater agricultural productivity, farther from public protected areas, and nearer other human features. Analysis of ownership and conservation status revealed sources of variation, with important differences between local and state government ownerships relative to non-governmental organizations (NGOs), and among U.S. Geological Survey (USGS) GAP program status levels. NGOs were more likely to have easements nearer protected areas, and higher conservation status, while local governments held easements closer to settlement, and on lands of greater agricultural potential. Logistic interactions revealed environmental variables having effects modified by social correlates, and the strongest predictors overall were social (distance to urban area, median household income, housing density, distance to land trust office). Spatial distribution of conservation lands may be affected by geographic area of influence of conservation groups, suggesting that multi-scale conservation planning strategies may be necessary to satisfy local and regional needs for reserve networks. Our results support previous findings and provide an ecoregion-scale view that conservation easements may provide, at local scales, conservation functions on productive, more developable lands. Conservation easements may complement functions of public protected areas but more research should examine relative landscape-level ecological functions of both forms of protection.

## Introduction

The cumulative effect of conservation decisions made by landowners, governments, communities, and funders is a landscape structure with particular ecological functions [1]. The result of myriad past land use decisions is written on the landscape [2, 3], and has been investigated by examination of existing patterns of distribution of ecosystems [4]. A recent trend is to study distributions and functions of conservation lands, including public conserved lands and private lands under conservation easements or other management-restricted status [5–8]. Understanding the geographic correlates of a distribution is prelude to uncovering causal relationships; we can attempt to uncover drivers of spatial distribution, as we would any natural or cultural phenomenon with a spatial component [9]. Doing so can help direct future conservation decisions especially if the finding is that spatial distribution is driven less by conservation goals, than by more proximal social or economic drivers. Building functional reserve networks relies on ability to site future easements and other management actions outside of public protected areas, for maximum long-term conservation benefit [10, 11].

The spatial distribution of conservation lands can tell us a great deal about their functions, as well as potential reasons for their establishment. Protected area distribution has been of conservation concern because of the historical tendency to site larger areas more remotely (e.g., “rocks and ice”), and the fear that more biologically productive and rich areas may be avoided due to their economic and social values [12]. Analyses have uncovered 1) spatial bias toward remote areas that face little threat [6], 2) lack of coverage of areas of highest species richness and endemism [13, 14], 3) incomplete representation of ecological processes such as disturbance [15], and 4) human encroachment and inability to enforce resource extraction [16, 17]. Conservation easements on private land are a more recent phenomenon and only over the last decade have mapped data become publically available to analyze their spatial distribution and potential conservation functions [18]. While a U.S.-focused conservation mechanism, easements have their analogs in other parts of the world, including conservation covenants in the United Kingdom and commonwealth [19], and conservation contracts for ecosystem services in the tropics [20]. Land use certification programs, community managed forests, and spatial planning and land use policies are widely employed, globally [21], and in some cases provide stronger conservation functions than do public protected areas. For example, a study of community managed forests in the tropics suggested that when local communities manage forests without top-down government control, the result is lower and less variable annual deforestation rates than in forested protected areas [8]. The rise in numbers of conservation easements in the U.S. indicates broad public support for private lands conservation and potential for those lands to contribute to biodiversity conservation, climate resilience, and social-economic services [5, 22, 23]. There are about 105,000 mapped easements, approximately 1/3 of the conservation lands in the U.S. (Protected Areas Database of the U.S. V1.3). Using such data, analyses may be performed at multiple spatial scales that can shed light on easement spatial distribution, environmental and social correlates of that distribution, and potential conservation functions.

Ecoregional conservation planning has been a cornerstone of spatial prioritization, as it seeks a scale relative to decision making that is both relevant to local decisions within relatively homogenous geographies, yet contains elements that can be considered, hierarchically through time and space, to the scale of continents, and the globe [24, 25]. Long the province of non-governmental conservation organizations [26], an effort has been recently launched in the United States to bring the tools of regional conservation to collaborative efforts of state and federal government agencies. This consists of 22 Landscape Conservation Cooperatives (LCCs) with multistate geographies following, more or less, ecologically defined regions [27]. The Appalachian LCC is a large, 15-state region encompassing a biologically rich (aquatics and terrestrial) area with a long history of human occupation and resource use [28]. Thirteen percent of the geography is in federal hands (primarily U.S. Forest Service; USFS andNational Park Service; NPS), a low percent is state lands (<2%), leaving roughly 85% privately owned and managed. An increasing level of energy extraction for coal and gas throughout the region, combined with pressure for amenity development (second homes, retirement homes, golf courses, services) near attractive features, and vulnerability of some ecosystems to rapid-onset climate change has focused renewed conservation attention on the region [29, 30]. Much of the new focus for building conservation landscapes that provide region-scale functions (e.g., climate resilience) is focused on where to site new projects on private lands [31].

While the National Conservation Easement Database (NCED) allows a continent-scale analysis, we chose to work at the regional level because it afforded the opportunity to assemble finer-scale datasets on environmental and social variables, and as an environmentally and socially cohesive area of the continent, it provides a means of interpreting results in light of environmental and social context. Specifically, our goals were to 1) describe the spatial distribution of conservation easements as a function of social and environmental correlates, 2) investigate spatial correlates for easements in different ownership and conservation status categories, 3) interpret spatial distribution relative to potential conservation functions, and 4) outline further research to elucidate landscape functions at multiple spatial scales.

## Materials and Methods

### Study Area

**Fig 1:**
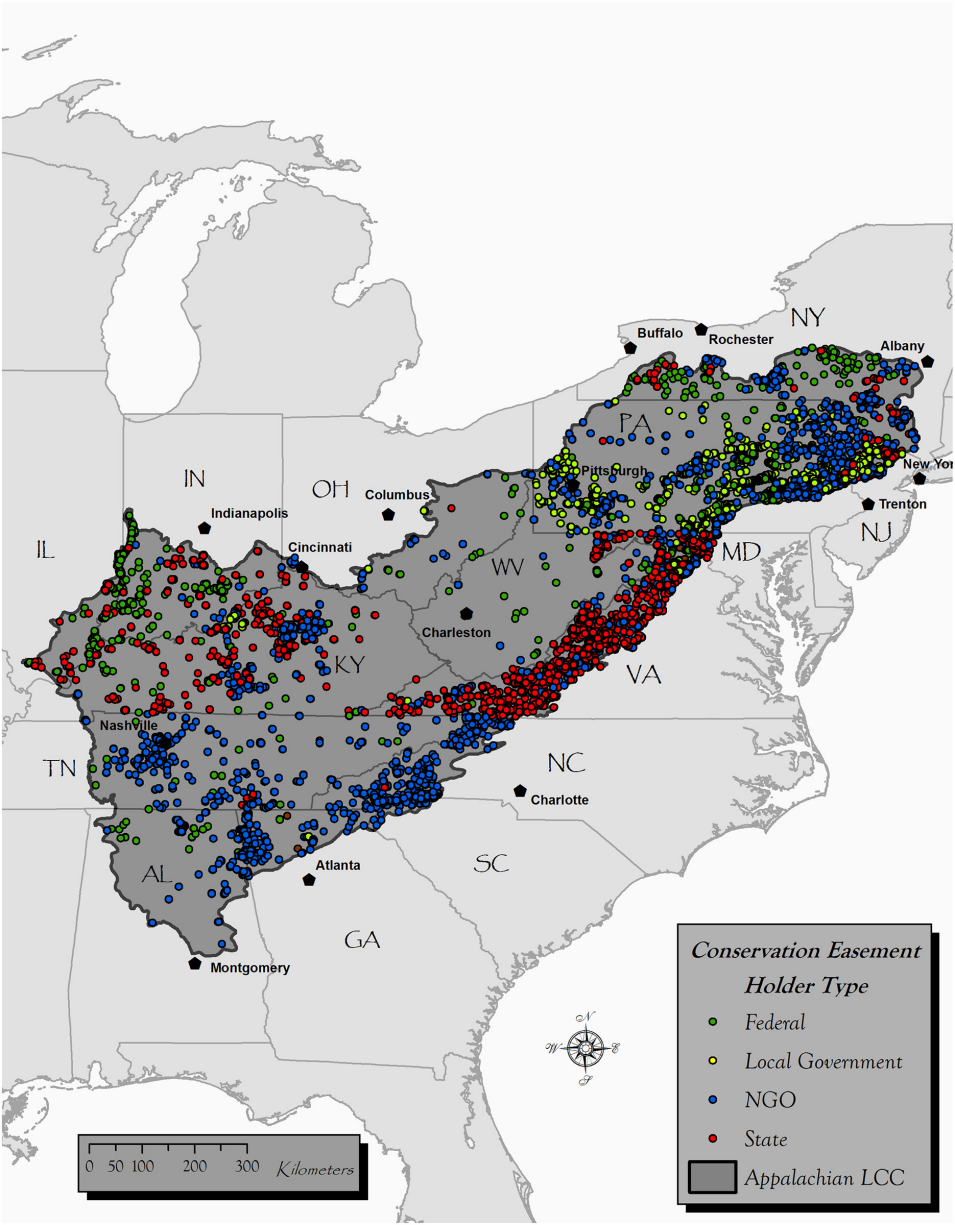
Spatial distribution of conservation easements within the study area (589,000 km^2^). Easement locations are categorized by 4 holder types.

The Appalachians are a useful region in which to test assumptions relative to the geographic proxies underlying spatial distribution of conservation lands. Thousands of years of human occupation, more than a century of intensive land uses and industry, a history of large-extent conservation evident in the establishment of 77,000 km^2^ of public lands and over 7,000 mapped conservation easements, rendered this region instructive as a conservation laboratory (Fig 1) [28, 32]. Topographically complex and biologically rich, the region encompasses ten of the highest peaks in eastern North America [33]. Within elevation gradients, landscape heterogeneity in vegetation cover is produced by surficial geology, aspect, landforms, and disturbance [34], and topography drives human settlement [35]. Westward from the Appalachian spine, regional forest patterns follow environmental-climatic gradients at broader spatial scales [36, 37]. Two of the 9 highest priority areas for new conservation action to protect endemism in the U.S., including the southern and central Blue Ridge mountains and watersheds, are Appalachian [13]. At 589,000 km^2^ the Appalachian LCC intersects part or all of 15 U.S. states (Fig 1). The coverage by public protected areas (~15%) is greater than that elsewhere in the eastern part of the U.S., but less than in the Western U.S. where some geographies have upwards of 50% coverage. Since Appalachia is a long-settled area with less remoteness than other areas of the world where protected areas are known to be “high and far”, we conducted simple tests to see if they followed roughly the same spatial distribution, for purposes of comparison with our easement results (α = 0.05). We found that public protected areas in Appalachia (N = 2,466) occurred at higher elevation (M = 387.5 m, SD = 219.1 m; *t* (2465) = 2.28, p = 0.01), and with lower crop productivity (M = 0.264, SD = 0.224) than random locations (N = 2,599) in unprotected areas (*t* (2465) = -15.6, p < .001).

### Sampling Design

We extracted records from the U.S. NCED, a public database containing shapefiles and tabular information for over 105,000 easements. This product is supported through a consortium of NGO and government partnerships. One of those partnerships is with the U.S. Geological Survey (USGS) GAP program, which assigns a conservation value to easements using standards that differ from the International Union for Conservation and Nature (IUCN). GAP status 1 is designated for areas under complete protection (e.g., no suppression of disturbance), GAP 2 areas allow some management (e.g., suppression of disturbance), GAP 3 areas allow some forms of extraction (e.g., localized mining), and GAP 4 areas have no ‘legal’ mandate of protection. The IUCN only recognizes GAP status 1 and 2 as ‘protected’ although GAP 1–4 easements all have some permanent protection from land cover conversion. Thus, GAP status 4 easements have conservation value by limiting development despite their GAP level assignment (Mitchel Hannon, personal communication, Dec. 24, 2014). Since easement data are controlled by thousands of local organizations (e.g., land trusts), assembly of spatial data in a grassroots, bottom up effort is fraught with staffing and privacy concerns resulting in an under-representation for some areas, and incomplete metadata.

The study sample included every easement within the Appalachian LCC that was attributed with the data needed for the analyses, and not completely contained in an ‘urban’ area as defined by U.S. Census (N = 4,813 of 7,449 total). Specifically, if an easement record included both easement holder and conservation level (GAP status) information, it was retained (S1 Table). Random sample points were generated within the easements, proportional to area (where larger easements contained more sample points), using a minimum inter-point distance of 100 m ($\bar{x}$ distance between points = 2.3 km). Minimum sampling distance was explored using experimental semivariograms to estimate the distance thresholds at which data were no longer autocorrelated. We then applied a heuristic of minimum spacing distance at a maximum of 70% of the spacing theoretically possible in order to sample the entire area. Points were generated in random unprotected locations while maintaining the same minimum sampling distance, but N was less than above due to deletions resulting from topology in the spatial data (N = 4,420). Due to the less restricted space, inter-point distances were somewhat greater ($\bar{x}$ distance = 5.6 km). Unprotected was defined as areas outside of mapped public protected areas and easements. Census-defined urban areas were excluded from the “unprotected” sampling due to the radically different landscape conditions they represent. Federally-held easements were excluded from the holder analysis due to very low numbers (<1% of mapped easements). We built a geographic database of environmental and social variables we hypothesized, based on the literature and our experience, would influence easement location. To examine the validity of our *a priori* thinking, we tested for differences of each variable between random points generated within easements and random unprotected locations. If we found significant differences between social and environmental variables for points inside versus outside easements, we could assume that they may be of value in predicting easement location.

### Data Description, Acquisition and Processing

For each random point we extracted elevation and the horizontal slope from the National Elevation Dataset [38]. We used the newly developed National Commodity Crop Productivity Index to describe soil productivity in regards to major crop growth (e.g., corn, soybeans, small grains, cotton) taking into account landscape and climate conditions [39]. Diversity of remotely-sensed land cover categories has been used as a surrogate for landscape-level ecological diversity (i.e., landscape-level habitat heterogeneity) [40–43], which we calculated from the National Land Cover Dataset (2010) using a moving neighborhood analysis and Simpson’s index [44]. We collated the most recent (2010) data for income and housing at the county and block levels from the U.S. Census then calculated density by housing units per hectare. We measured straight-line distances between all sample points and the nearest feature of the following variables: roads, public protected areas, urban areas, water body, and land trust (Table 1).

**Table 1:** Predictor variables, aliases used, source of data, product name or derived product, spatial resolution, and year released to public.

Untransformed Predictors	Alias	Source	Product	Spatial Resolution	Year
**Elevation (m)**	**Elevation**	U.S. Geological Survey	National Elevation Dataset	30 m	Continuous
**Slope (d)**	**Slope**	Author	Distance to Horizontal	30 m	2014
**NCCPI (0–1)**	**Productivity**	U.S. Dept. Agriculture	National Crop Productivity Index	90 m	2013
**Distance to Water Body (km)**	**Water**	U.S. Geological Survey	National Hydrologic Dataset	Vector	2012
**Distance to Protected Area (km)**	**Protect**	U.S. Geological Survey	Protected Areas Database—US	Vector	2014
**Landcover Diversity**	**Diversity**	U.S. Geological Survey	Landcover Diversity	1 km	2002
**Distance to Urban (km)**	**Urban**	U.S. Census Bureau	Urbanized Areas	Vector	2010
**Distance to Road (km)**	**Road**	U.S. Census Bureau	Tiger/Line	Vector	2014
**Median Income (Co)**	**Income**	U.S. Census Bureau	2010 Census—American Fact Finder	County	2010
**Housing Density (Co)**	**Density**	U.S. Census Bureau	2010 Census—American Fact Finder	County	2010
**Median Income (block)**	**Block Income**	U.S. Census Bureau	2010 Census—American Fact Finder	Census Block	2010
**Housing Density (block)**	**Block Density**	U.S. Census Bureau	2010 Census—American Fact Finder	Census Block	2010
**Distance to Land Trust (km)**	**Trust**	The Land Trust Alliance	Directory of Land Trusts	Vector	2014

### Model Development

**Fig 2:**
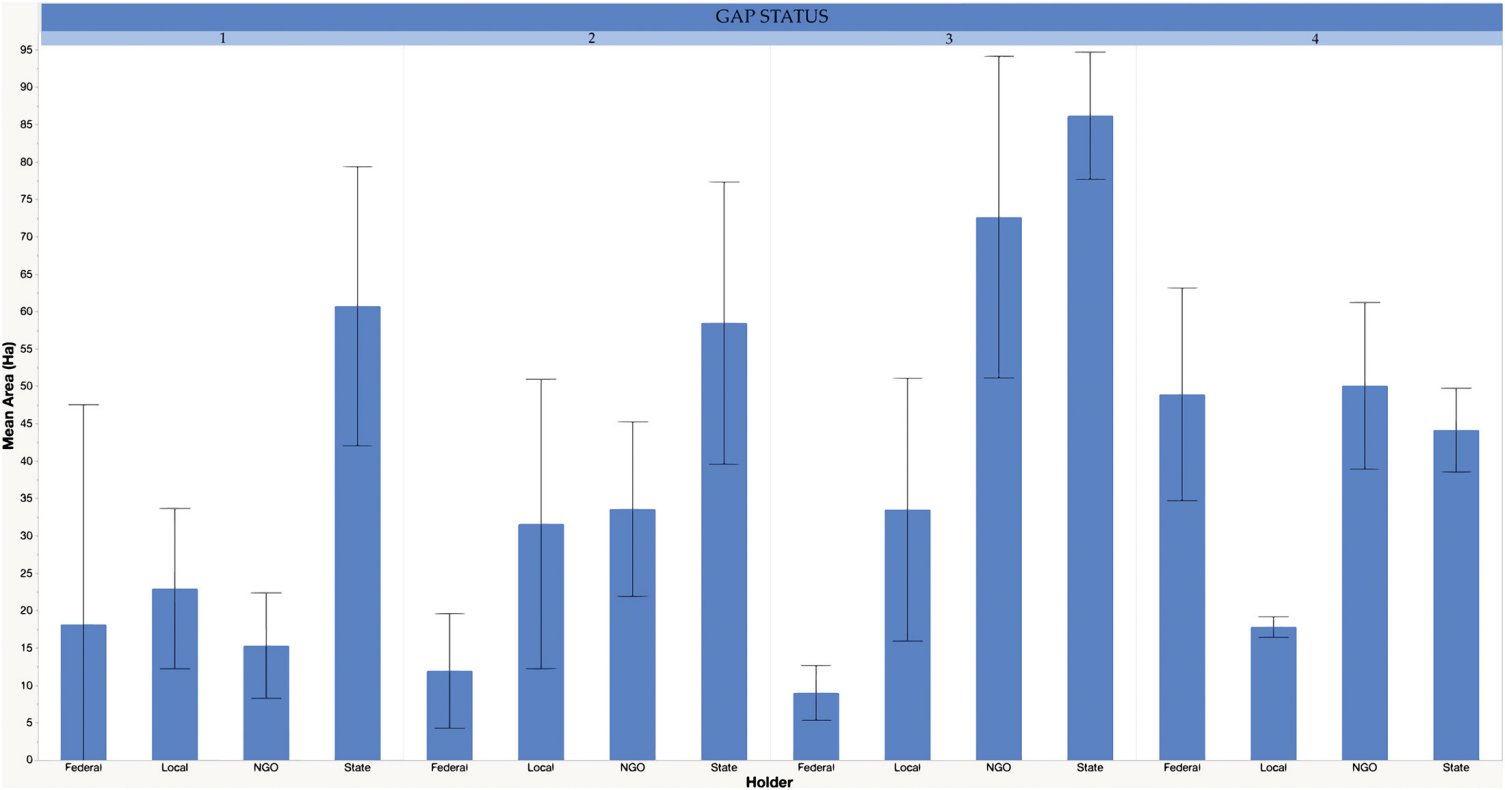
Mean area (ha) and numbers (N) of conservation easements by easement holder in each GAP level (1–4) within the Appalachian LCC. There are 363,000 ha of easements in the region; 87% of the easements are in the multiple-use GAP categories 3–4. The relatively high mean area of Federally-held easements in GAP 4 is due to the existence of a few, large holdings.

We used logistic regression with interactions to model spatial relationships of easements and social/environmental variables. First, we compared conservation easement samples with random unprotected areas in order to examine location bias in relation to the explanatory variables. We used a mixed-direction stepwise logistic model selection approach for considering the best second-order models. This approach facilitated the examination of parameter interactions, in particular social and environmental interactions (e.g., distance to urban areas and elevation gradients) that moderate the covariates’ ability to predict the dependent variable. Second, we examined how the level of conservation for an easement and top easement holders (i.e., those with a large enough sample which were state, NGO, local government, and not federal; Fig 2) can be predicted by the explanatory variables using a main effects, multinomial logistic approach.

We carried out all statistical analyses using the statistical program R (v. 3.1.0) and where needed, used α = 0.05. For binary logistic regression we used the generalized linear model library (glm2) and for multinomial analysis we used the Feed-forward Neural Networks and Multinomial Log-Linear Models library (nnet). We completed cross-validation using the Data Analysis and Graphics library (DAAG). Finally, we conducted Durbin-Watson tests for autocorrelation (bootstrapped 1,000) of model residuals and examined variance inflation factors to test for multicollinearlity. We found no significant violation of test assumptions.

### Model Comparison, Validation, and Relative Importance

To compare our logistic models we used the Display and Analyze ROC Curves (‘pROC’) package [45]. With bootstrapping, we produced 1,000 stratified areas under the curve (AUC) samples for each model and computed their differences:

$$D=\frac{AUC1-AUC2}{s}$$

where s = standard deviation of the bootstrap differences for sensitivity and specificity. We then compared D to the normal distribution for hypothesis testing. In order to test the accuracy of our non-linear models we performed repeated k-folds cross validation. This technique randomly divides observations into k groups of roughly equal size while fitting the model with k-1 groups and measuring prediction accuracy of the remaining group [46]. This is repeated k times (folds) until each group is predicted once and used to fit the model k-1 times. We then repeated (N = 100) and used the mean of this output to decrease the dependence of the estimate on the initial group partitions.

In addition, we wanted to determine if social predictors were relatively more important in the model than environmental predictors. We tested relative importance between these two groupings using the Relative Contribution of Effects in a Regression Model package (Relimp). Relative importance is measured using a ratio of the variances of contributions by the two groups to the log-odds of predicting an easement location [47].

## Results

### Conservation Easement Location

The predictors had robust *a priori* support: all hypothesized environmental and social variables with the exception of census block housing density differed in their values when compared inside easements versus random (Table 2). Logistic regression analysis of easement locations relative to social and environmental predictors revealed that, when all easements were considered together regardless of ownership or conservation status category, the odds of finding a conservation easement were greater closer to urban developments, major roads, in areas with greater crop productivity potential, slighter slopes, and in lower-elevation settings that had greater landscape heterogeneity. A first order model with all predictors was useful for predicting easement locations (missclassification rate = 26%), yet exploratory analysis indicated interactions were operating on the predictors and including these would improve model performance. The model including interactions adequately fit the data with a significant chi-square test statistic (χ^2^ (34, *N* = 9232) = 3738, *p* = < 0.001), a rejection of ‘lack of fit’ using the Hosmer-Lemeshow test (p > χ^2^ = 0.87), and correctly predicted the occurrence of an easement 77.4% of the time.

**Table 2:** Comparison of mean predictor variable values within conservation easements versus random locations in unprotected areas within the Appalachian LCC.

Untransformed Predictors	Mean Value		T-test results		
	Random	Easement	p-value	Mean Difference	95% CI
**Environmental**					
Elevation (m)	372.44	332.30	< .001	40.28	31.59–48.97
Slope (degrees)	10.12	7.91	< .001	2.21	1.90–2.52
NCCPI (0–1)	0.34	0.39	< .001	0.048	0.038–0.058
Distance Water Body (km)	0.45	0.49	< .001	0.044	0.026–0.062
Distance Protected Area (km)	6.81	4.48	< .001	2.33	2.13–2.54
Landcover Diversity	92.38	104.68	< .001	12.3	10.16–14.45
**Social**					
Distance Urban Area (km)	13.72	10.97	< .001	2.76	2.40–3.11
Distance Road (km)	16.72	12.24	< .001	4.79	3.98–4.98
Median Household Income (Co)	40,850.27	50,367.95	< .001	9,516	9,103–9,930
Housing Density (Co)	0.25	0.57	< .001	0.31	0.287–0.340
Median Household Income (census block)	45,189.47	59,841.53	< .001	14,652	13,873–15,431
Housing Density (census block)	0.25	0.27	0.323	0.019	0.019–0.058
Distance Land Trust (km)	52.46	24.24	< .001	28.22	26.88–29.56

P-values refer to t-tests and effect sizes (mean difference) and confidence intervals of the difference are given.

**Fig 3:**
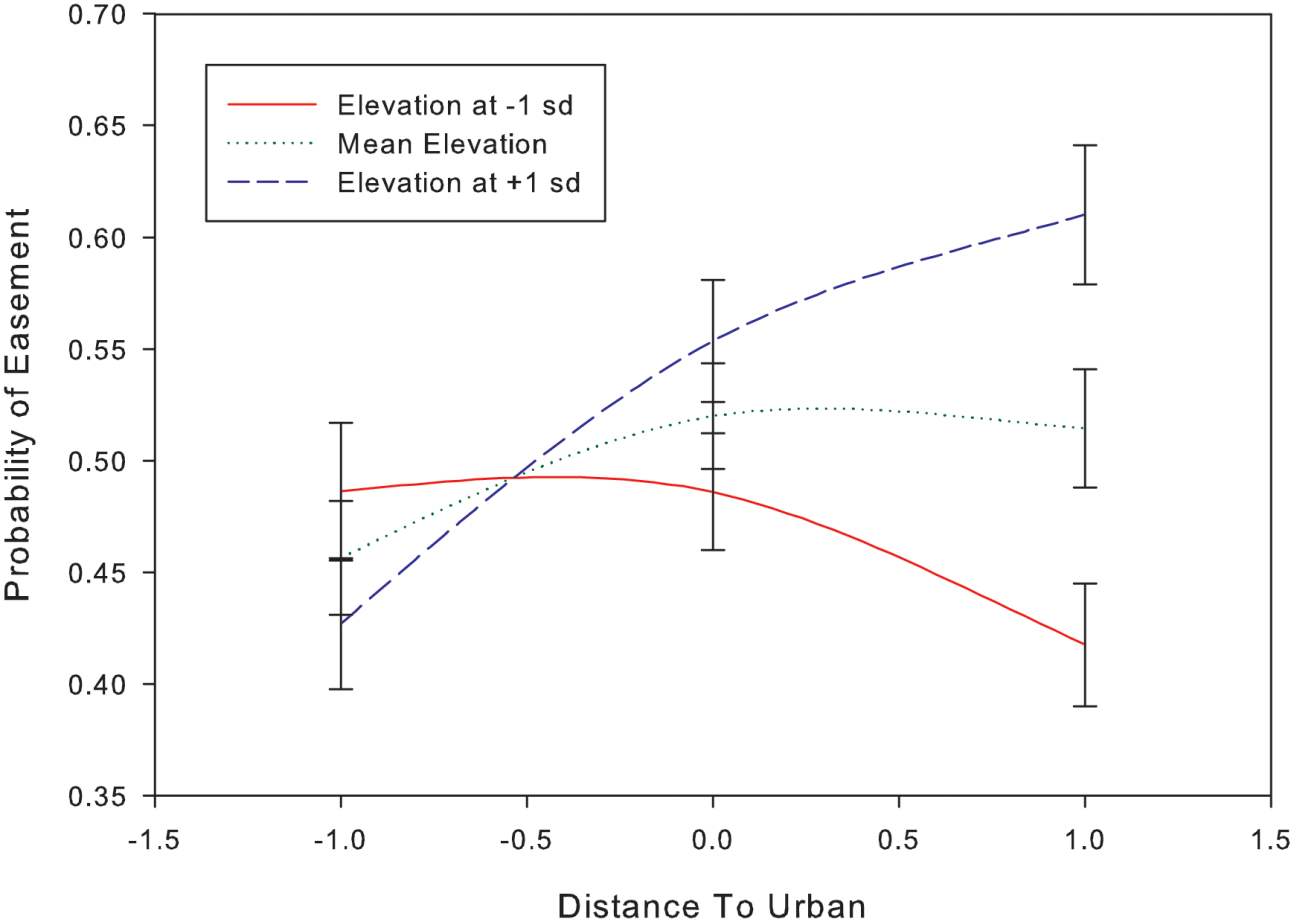
The interaction effect of elevation on distance to urban for predicting conservation easement location. As elevation departs from its mean (± 1SD), the effects of distance to urban area on probability of easement location becomes stronger.

Interactions can be difficult to interpret in logistic regressions unless one is familiar with the use of odds ratios [48]. To illustrate, we use the example of the interaction between distance to urban areas, and elevation (Fig 3; S2 Table). If elevation was near its mean value, it had only a moderate effect, and urban was not a good predictor of easement location. However, if elevation was lower than its mean then distance to urban area became a more important effect, and being closer to an urban area increased the odds of being in an easement. Conversely, if elevation was above its mean, then increasing distance to urban area led to increased odds of being in an easement. The degree to which distance to urban improves easement prediction given a one standard deviation increase in elevation can be seen in the odds ratio in Appendix A (i.e., multiplicative factor = 1.29 ± 0.07 or 29%).

**Fig 4:**
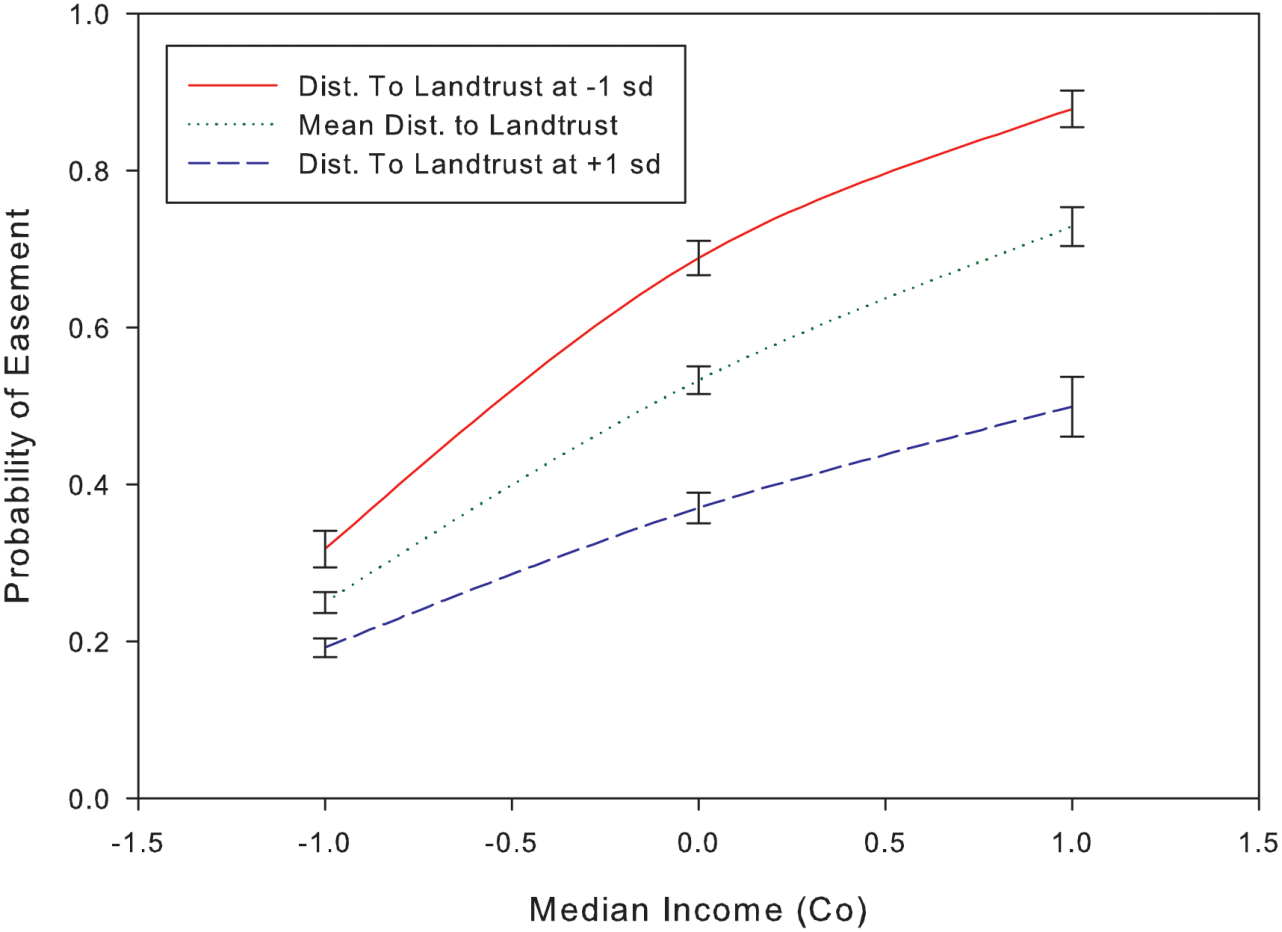
The interaction effect of distance to nearest land trust address on median county income for predicting conservation easement location. The closer an easement was to a land trust, the greater the positive effect of income; easement locations farther from a land trust were associated with higher incomes, but the effect was less.

A similar strength interaction effect occurred between median county income and distance to the address of the nearest land trust. For this interaction, increasing income was positively associated with location of easements but distance to the nearest land trust affected the degree of this effect (Fig 4; S2 Table). The multiplicative factor describing the impact of increasing the distance to land trust, on the utility of income to predict easement location was 0.72 (±0.08) or -28%. The closer an easement was to a land trust, the greater the positive effect of income; easement locations farther from a land trust were associated with higher incomes, but the effect was less (odds ratio closer to 1). Most significant interaction effects we observed were either between two social predictors or between one social and one environmental predictor (as above).

### Model Comparison, Validation, and Relative Importance

We used the top six environmental and social predictors to assess relative importance between the groups in a main effects model. If the two groups contributed to the variation in the log-odds of being in an easement equally, the ratio difference would equal zero (i.e., X/Z = 1). We found that social predictors explain more variation (ratio = 1.37, p = < 0.001) in predicting easements as a group than the environmental variables we measured.

The top second-order model included census block data (housing density and median income). However, many of the easements were larger than single census blocks, which could confound the variables. Thus, we omitted block-scale data for this analysis and compared the top main effects model with our chosen second-order model to determine if interaction terms significantly improved model performance. Area under the curve for the main effects model was 0.821 (±0.008) and the second-order model AUC was 0.845 (±0.008). The bootstrapped differences (N = 1000) resulted in a value of D = -12.7 leading us to reject the null hypothesis that the differences between the two models equaled zero (p = < 0.001). Repeated k-folds cross validation provided confidence for our model with predictive accuracy of (0.7704) which only differed from the training data (0.7736) by 0.42%.

### Level of Conservation within Easements

Of the total easements in our sample, 64% (N = 3061) had been assigned a conservation level by the NCED. Of these, 3% were GAP 1, 7% GAP 2, 33% GAP 3, and 57% GAP 4. All explanatory variables were useful in predicting GAP status except distance to water (χ^2^ (36, *N* = 3061) = 1479, p = < 0.001). We rejected the null hypothesis of lack of fit using the Hosmer-Lemeshow test (p > 1.0), and attained a prediction accuracy of 74.6% with GAP 2 easements being the most poorly fit. The most useful predictor of GAP level was distance to nearest land trust. Specifically, a one standardized unit change in this predictor (closer to land trust office location) increased the odds of being in either GAP 3 (9%) or GAP 4 (46%) relative to GAP 1 (Table 3), indicating that easements with a lower protection status were geographically closer to the physical address of a land trust. When increasing the distance to road or urban, the odds of being in a higher GAP status easement increased (88% and 92% for GAP 3 and 4 relative to GAP 1). Increasing elevation was strongly associated with easements that allowed for greater management as the odds increased for GAP 3 (247%) and GAP 4 (97%) relative to GAP 1. The most useful environmental predictor for this analysis was distance to nearest public protected area. Occurring closer to one of these areas increased the odds of being in GAP 2 (64%) and GAP 4 (41%) relative to GAP 1. Other useful predictors across multiple GAP levels included census block-level income, county housing density, and slope. The least useful predictors in the model (odds ratios closest to 1) were crop productivity index, census block housing density, and county-level median income. Lastly, although social predictors as a group were more important than environmental ones at every GAP level, they become relatively less dominant with increasing conservation level (from a ratio of 1.52 to 1.25 between GAP 4 and GAP 1, respectively).

**Table 3:** Relationships among easement holder categories (A), conservation status categories (B), and their associations with environmental and social spatial variables.

	**Road**	**Urban**	**Income (Co)**	**Density**	**NCCPI**	**Elev**.	**Slope**	**Income (B)**	**Density (B)**	**LandTrust**	**Protect**	
**A**	**Relative to NGO (Reference Category)**											
Local	14.0	-35.7	2.2	7.9	74.6	-69.5	14.3	-98.8	-48.9	14.9	55.3	
State	-7.7	-24.3	20.7	-39.9	7.8	19.7	26.7	22.7	-44.4	202.4	35.9	
	**Road**	**Urban**	**Income (Co)**	**Density**	**NCCPI**	**Elev**.	**Slope**	**Income (B)**	**Density (B)**	**Landtrust**	**Protect**	**Diversity**
**B**	**Relative to Gap 4 (Reference Category)**											
Gap 3	-28.5	-33.2	-15.9	-66.5	19.9	76.0	42.4	38.8	-40.8	70.8	91.4	33.4
Gap 2	37.4	-36.1	-2.0	-1.3	-25.4	-16.1	17.1	64.5	1.7	134.4	-38.4	18.4
Gap 1	-47.1	-48.0	-69.0	-16.9	-1.6	-49.3	48.3	51.6	29.4	86.7	68.8	-9.8

Values are percent change in the odds of moving from the reference category to the test category (groups A or B) for a one-unit increase in each variable. The effect size and direction can be interpreted as a greater, negative % change in odds means as the value of the variable decreases (e.g., closer to feature, or lower in elevation), the odds of being in the reference category decrease relative to the odds of being in the test category, and the reference category would be farther from, or lower than, the test. Federal holders not included in analysis A due to their very small numbers. Only predictors that were significant in top models are shown (e.g., Diversity in Analysis B).

### Easement Holder

We predicted easement location for the three most frequent ownership types, which collectively account for 99% of the easements sampled (NGO: 40%, state: 28%, and local government: 32%), using the same explanatory variables as in previous analyses (Table 1). Predictors that were not significant in the top model were landcover diversity and distance to nearest water body. The difference between the full and reduced model was significant, χ^2^ (22, *N* = 4742) = 4496, *p* = < 0. 001) and we rejected the null hypothesis of the Hosmer-Lemeshow test for ‘lack of fit’ (p > χ^2^ = 1.0). Although the overall classification accuracy was moderate (73%), the AUCs for both local and state government easements were high (0.96 and 0.90, respectively).

We observed a striking trend towards NGOs holding easements closer to existing public protected areas, on gentler slopes, with less crop productivity, located farther from urban areas, and lower median county income. We observed that county and municipal governments, relative to NGOs administer easements in census blocks with lower median incomes but on land with higher crop productivity, at lower elevations. Meanwhile state governments hold easements that are higher in elevation on steeper slopes with lower county housing density and higher median county income, and much farther from land trust offices, relative to NGOs.

## Discussion

Land use decisions on private lands are among the most important for conservation given that coverage by public protected areas falls short of global targets, and their spatial distributions systematically exclude ecological settings [12, 14, 49–51]. Understanding the environmental and social characteristics associated with spatial distribution of conservation actions can illuminate their current conservation functions, and direct locations of future actions for maximum conservation benefit [18, 23, 52]. We found that conservation easements have a spatial distribution driven by interacting social and environmental factors and that social variables were the most strongly associated with easement location. Overall, the odds of finding an easement were greater closer to urban areas and at lower elevations, in areas of greater housing density and median incomes, closer to roads and with greater landscape-level habitat heterogeneity, in areas of greater crop productivity, and farther from public protected areas. This tendency towards “low and near” for conservation easements stands in marked contrast with the global trends in spatial distribution of public protected areas [6]. Compared to random, unprotected locations, many easements in Appalachia occur at lower elevations, nearer human settlement, and on higher value agricultural lands. Appalachian public protected areas roughly followed the global trends, being at higher elevations and on less productive lands, than random, unprotected areas. Easements may complement spatial distribution of protected areas and may represent systems underrepresented by traditional protected areas. Our easements results align, at a greater spatial extent, with those from a more local geography (9-county area) that locations of easements were nearer human occupation, captured more open and agricultural ecosystems, and complemented conservation functions of public protected areas [18]. A trade-off may be that conservation function of easements thus scattered in human-dominated low-lying areas of the mountains is compromised by proximity to urban areas, and fragmentation. Further research on the landscape-level functions provided by easements in the context of the reserve network and land use matrix would illuminate the costs and benefits of current distribution.

When we looked more closely at ownership category (i.e., easement holder) and conservation status level (i.e., GAP status 1–4) we found variation not seen in the above, general trends for all easements in the sample. Local and state-held government easements were likely to be closer to urban areas, and higher in housing density than the NGO-held easements. NGO easements were likely to be much higher in elevation than local government easements, but only slightly lower in elevation than state held easements suggesting that states and NGOs may be following similar rules in where they choose to establish conservation easements. Interestingly, state-held easements were likely to be much farther (202% change in odds; Table 3) from the address of a land trust than NGO-held easements. It is not surprising that NGOs would site easements closer to land trust offices than would state or local government-owned easements. While there is not empirical support yet for how attitudes influence spatial distribution of easements, public attitudes about wind farms are strongly related to proximity of the dwelling to the windfarm [53]. The strength of the % difference in odds was so great between state owned and NGO owned easements for this variable that we believe there might be a phenomenon related to proximity to where conservation practitioners live and work, and its effect on easement location, worth further study. Location bias relative to ease of access is well known in field biology, where species location samples often cluster near home addresses of field observers, and along travel routes [9].

Our result that NGOs were likely to have had easements much nearer existing protected areas, than were either local or state governments is consistent with other studies. Employees of The Nature Conservancy (TNC) surveyed for information about a sample of their easements reported that 79% of their sample were adjacent to protected areas, suggesting that NGOs with an explicit, large-extent conservation mission may be spatially prioritizing establishment in the context of an existing protected areas network [10]. Our study supports their findings by examining the spatial distribution of the easements themselves, and differences in spatial distribution among categories of easement holder (Table 3). Much work remains to examine, within holder categories (e.g., within NGO types and missions), how spatial characteristics of decisions may differ. As discussed below, building out of holder attribute data within the NCED and other public protected area databases will allow finer-scale examinations of social drivers. One of the more interesting effects we noted relative to complementarity in conservation planning was the strong positive association of local government-held easements within areas of greater crop productivity; the area of locally-held easements was moderate compared to that of other holders (Fig 2), yet their spatial locations may be such that they capture ecosystem types typically underrepresented in regional networks of conservation lands [12, 54]. Based on available attribute data, we don’t know if easements were established for agriculture e.g., under “farmland-forever” type programs [23]. Easements established for agriculture can provide different biodiversity functions, for example maintaining lower resistance in land use matrices relative to developed lands, and positively influencing connectivity at greater spatial scales [55, 56].

In every comparison there were scale effects evident for two variables reflecting human settlement and economics (i.e., median household income and housing density). Census blocks—the minimum spatial units for which census data are quantified—vary greatly in area, with smaller blocks occurring nearer urban areas, and larger blocks in rural areas. When census block data are summarized for the county level (e.g., “Income Co”, “Density Co”) the effects in the regression are different than when left at the census block scale. For example, locally held easements are much more likely (99%) to be in lower income areas than NGO held easements when examined at the block level, but slightly more likely to be in higher income areas when examined at the county level. Similar scale effects were noted in a study of land use change in this region, in that county-level aggregate data showed little change over time, while spatial distributions of settlement within counties changed substantially [35]. For easement distribution, an explanation may be found in the cost of land, and the tendency for opportunity to drive easement acquisition with smaller, lower income blocks being likely to be nearer easements held by local governments, but those are likely to be in counties with average or slightly greater income levels, overall. A fine scale analysis of these associations and causes is beyond the scope of this paper yet could be important for understanding how our understanding of spatial pattern is influenced by grain size of predictor variables [57].

Our study is limited in other ways. On a fundamental level, we don’t know for what conservation purposes the easements were established in the sense that for this great a spatial extent, there is no single database providing information on the conservation goals, as might be garnered from deeds, monitoring plans, or opinions of land trust staff [5]. The NCED provides insight into their purpose via conservation status (GAP 1–4), and type and name of easement holder, yet these metadata are incomplete (65% of the NCED had ownership-status metadata we needed for this analysis). Matching resolution of data to conservation objectives is an important aspect of conservation planning [58]. Finer scale datasets are available for more local areas (e.g., tax parcel databases and deeds at the county level), but collating these to a region or nation is a massive undertaking. The NCED is relatively coarse in its representation of the easement phenomenon, but is the only opportunity to study patterns in easement location at the ecoregion scale, an extent useful for conservation planning [26]. We had to omit nearly 35% of mapped easements in the NCED from the sample due to missing attribute data. There is the danger of systematic bias in such a non-random sample, which is difficult to assess when key attributes are missing. For one calculated metric (easement area), there was no statistical difference between omitted and included data (t = 0.2967, df = 7447; p = 0.7). Future studies should be aware of potential differences between features with and without data on key attributes. We omitted federal easements from the holder analysis because of their low numbers (<1%). In regions where federally held easements are more prevalent, they should be included. We did not use State as a variable because the proportion of areas of states represented included low values (2–100%). Ecoregion-based analyses are limited in their ability to make inference based on political units, because study area boundaries do not follow political boundaries. Finally, we recognize that new spatial data are continually updated and released, for example new versions of PADUS, NCED, and species richness data [13].

Importantly, we have yet to analyze the specific conservation functions of easements. This is an emerging area of research including comparison of conditions inside and outside of easements, for which we provided an initial investigation in this paper. Also important is how easements provide landscape level functions relative to distribution of conservation core areas, buffers, and corridors at varying spatial resolutions. Our results provide an examination of geographic correlates of the observed distribution at a larger extent and bigger sample of easements than previous studies, a prelude to more intensive studies of landscape-level function at the region scale. Finally, a limitation of our study was that we did not specifically compare the spatial distribution of easements to that of public protected areas. Our study was focused on statistical analysis of correlates of distribution of easements in different ownership and conservation level categories, but to investigate general patterns in our region, we conducted a brief comparison of protected area distribution with random, unprotected points. The result that protected areas in the region generally followed global trends being higher in elevation and in areas of lower crop productivity than random is enough to satisfy us that there are no big differences in pattern in this region. Future research will examine more specifically conservation function of easements versus public protected areas in light of regional representation of biodiversity and role in habitat connectivity. A strong set of reserve selection and habitat connectivity metrics can be employed to assess how alternative land and water conservation mechanisms (e.g., community forests, conservation easements), relative to public protected areas, represent regional diversity and provide connected cores in climate space [56, 59, 60].

Conservation on private lands takes many forms, each approach reflecting national histories of land ownership and government involvement in land use. Perhaps because of the tensions between rights of property ownership and oversight by federal and state governments in the U.S., easements have become popular, seen as a win-win result trading some land use rights away in return for payments or easing of tax burdens [61]. In the U.S., conservation easements are unevenly distributed and as of yet do not make significant, country-wide contributions to capturing patterns of species richness and endemism [13]. In other parts of the world, community forests, environmental certifications for timber and food production, and government-based spatial planning and regulation demonstrate that there is a range of approaches to limiting resource exploitation in exchange for conservation values [21, 62, 63]. Regardless of the conservation mechanism employed, the spatial distribution of these actions relative to the rest of the protected areas network can potentially have a large impact on the landscape-level functions provided that can, in turn, influence how well conservation goals are achieved [11, 13]. Our study shows that there are both social and environmental geographic proxies underlying the distribution of conservation easements, resulting in a landscape pattern of easements that may deliver unique conservation functions. We found that studying interactions among variables provides better insight into drivers for where conservation lands are established, than considering main effects only. More research is needed to determine what functions easements perform locally, and how those may complement functions performed at greater extents in the spatial hierarchy.

## Supporting Information

S1 Table Conservation easements in NCED that were removed from our analysis (N = 2636 removed out of 7,449). * Polygons with topological errors, specifically vector drawing errors that can result in them being counted twice.** Easement records that contained incomplete or contradictory data between Easement holder type and Ownership type.
(DOCX)

S2 Table Interaction results from binary logistic regression of easements versus random unprotected locations. An odds ratio > 1 indicates a positive relationship with the dependent variable, close to 1 indicates weak relationship, and <1 indicates negative. Percent change indicates the strength of the relationship (negative or positive) with a one standardized unit increase in the predictor term. These relationships are summarized by their absolute percent change (listed in descending order). The full model results are: probability of being in an easement = 0.246 + (0.086 *house) + (0.060 *road) + (-0.020 *slope) + (0.003 *water) + (0.123 *diversity) + (0.268 *elevation) + (1.157 *income) + (-0.862 *trust) + (0.158 *nccpi) + (0.151 *urban) + (-0.317 *protect) + (-0.190 *income *income) + (-0.121 *road *road) + (-0.137 *urban *urban) + (-0.079 *nccpi *nccpi) + (0.105 *diversity *diversity) + (0.255 * urban *elevation) + (0.327 *income *trust) + (0.287 *income *density) + (-0.261 *elevation *density) + (0.171 *urban *density) + (-0.201 *income *protect) + (0.158 *road *diversity) + (-0.176 *urban *protect) + (-0.174* slope *density) + (0.128* density *diversity) + (0.124 *income *slope) + (-0.140 *diversity *protect) + (-0.132 *road *density) + (0.109* slope *diversity) + (-0.122 *trust *protect) + (-0.099 *urban *nccpi) + (0.088 *water *slope) + (0.067 *water *nccpi). See Table 1 for full variable names.
(DOCX)
