# Supplementary material for: Interacting Social and Environmental Predictors for the Spatial Distribution of Conservation Lands
Source: PLoS One. 2015 Oct 14;10(10):e0140540. doi: 10.1371/journal.pone.0140540 (PMC4605775; doi:10.1371/journal.pone.0140540)
Supplement: S1 Table — * Polygons with topological errors, specifically vector drawing errors that can result in them being counted twice.** Easement records that contained incomplete or contradictory data between Easement holder type and Ownership type. (DOCX) [file pone.0140540.s001.docx]

| **Reason for omitting record from analysis** | **Count** | **Count** |
| --- | --- | --- |
| Missing Holder Attribute |  | 901 |
| Occurred in Urban Area |  | 273 |
| Vector Drawing Errors * |  | 841 |
| Contradictory Metadata ** |  | 159 |
| Missing Gap Status Attribute by Holder |  | 462 |
| NGO | 291 |  |
| Federal | 80 |  |
| Local | 9 |  |
| State | 70 |  |
| Unknown | 12 |  |
| **Total** | **462** | **2636** |

**S1 Table. Conservation easements in NCED that were removed from our analysis (N = 2636 removed out of 7,449).** * Polygons with topological errors, specifically vector drawing errors that can result in them being counted twice.** Easement records that contained incomplete or contradictory data between Easement Holder type and Ownership type.
