# Supplementary material for: Interacting Social and Environmental Predictors for the Spatial Distribution of Conservation Lands
Source: PLoS One. 2015 Oct 14;10(10):e0140540. doi: 10.1371/journal.pone.0140540 (PMC4605775; doi:10.1371/journal.pone.0140540)
Supplement: S2 Table — An odds ratio > 1 indicates a positive relationship with the dependent variable, close to 1 indicates weak relationship, and <1 indicates negative. Percent change indicates the strength of the relationship (negative or positive) with a one standardized unit increase in the predictor term. These relationships are summarized by their absolute percent change (listed in descending order). The full model results are: probability of being in an easement = 0.246 + (0.086 *house) + (0.060 *road) + (-0.020 *slope) + (0.003 *water) + (0.123 *diversity) + (0.268 *elevation) + (1.157 *income) + (-0.862 *trust) + (0.158 *nccpi) + (0.151 *urban) + (-0.317 *protect) + (-0.190 *income *income) + (-0.121 *road *road) + (-0.137 *urban *urban) + (-0.079 *nccpi *nccpi) + (0.105 *diversity *diversity) + (0.255 * urban *elevation) + (0.327 *income *trust) + (0.287 *income *density) + (-0.261 *elevation *density) + (0.171 *urban *density) + (-0.201 *income *protect) + (0.158 *road *diversity) + (-0.176 *urban *protect) + (-0.174* slope *density) + (0.128* density *diversity) + (0.124 *income *slope) + (-0.140 *diversity *protect) + (-0.132 *road *density) + (0.109* slope *diversity) + (-0.122 *trust *protect) + (-0.099 *urban *nccpi) + (0.088 *water *slope) + (0.067 *water *nccpi). See Table 1 for full variable names. (DOCX) [file pone.0140540.s002.docx]

| **Predictor Interactions** | **Estimate** | **P-value** | **Odds Ratio** | **CI 2.5%** | **CI 97.5%** | **%Change** | **Abs % Change** |
| --- | --- | --- | --- | --- | --- | --- | --- |
| urban*elevation | 0.25486959 | <0.001 | 1.2902933 | 1.2190239 | 1.3666015 | 29.029335 | 29.029335 |
| income*trust | -0.3268068 | <0.001 | 0.7212231 | 0.6449837 | 0.8057654 | -27.877695 | 27.877695 |
| income*house | -0.2874182 | <0.001 | 0.7501979 | 0.6873748 | 0.8207341 | -24.980208 | 24.980208 |
| elevation*house | -0.2607756 | <0.001 | 0.7704538 | 0.7051978 | 0.8409542 | -22.954624 | 22.954624 |
| urban*house | 0.17100672 | <0.001 | 1.1864987 | 1.0776684 | 1.3083481 | 18.649873 | 18.649873 |
| income*protect | -0.2006439 | <0.001 | 0.8182037 | 0.7629107 | 0.8770745 | -18.179629 | 18.179629 |
| road*diversity | 0.15750324 | <0.001 | 1.1705846 | 1.106228 | 1.2391632 | 17.058455 | 17.058455 |
| urban*protect | -0.1756393 | <0.001 | 0.8389205 | 0.7867354 | 0.8938376 | -16.107945 | 16.107945 |
| slope*house | -0.1738727 | <0.001 | 0.8404039 | 0.7740615 | 0.911715 | -15.959613 | 15.959613 |
| house*diversity | 0.12781908 | <0.001 | 1.1363474 | 1.063389 | 1.2143598 | 13.63474 | 13.63474 |
| income*slope | 0.1243957 | <0.001 | 1.1324639 | 1.0515511 | 1.2227518 | 13.24639 | 13.24639 |
| diversity*protect | -0.1403743 | <0.001 | 0.8690329 | 0.8205318 | 0.9204491 | -13.096706 | 13.096706 |
| road*house | -0.1318805 | <0.001 | 0.8764458 | 0.8021312 | 0.95599 | -12.355423 | 12.355423 |
| slope*diversity | 0.10872505 | <0.001 | 1.1148558 | 1.0558089 | 1.177738 | 11.485578 | 11.485578 |
| trust*protect | -0.1216834 | 0.002 | 0.8854286 | 0.8195468 | 0.9545355 | -11.457136 | 11.457136 |
| urban*nccpi | -0.0992844 | 0.002 | 0.9054852 | 0.8498383 | 0.96455 | -9.451483 | 9.451483 |
| water*slope | 0.08763082 | 0.003 | 1.0915851 | 1.0300184 | 1.1570741 | 9.158506 | 9.158506 |
| water*nccpi | 0.06701083 | 0.008 | 1.0693071 | 1.0175329 | 1.1241714 | 6.930706 | 6.930706 |

**S2 Table. Interaction results from binary logistic regression of easements versus random unprotected locations.** An odds ratio > 1 indicates a positive relationship with the dependent variable, close to 1 indicates weak relationship, and <1 indicates negative. Percent change indicates the strength of the relationship (negative or positive) with a one standardized unit increase in the predictor term. These relationships are summarized by their absolute percent change (listed in descending order). The full model results are: probability of being in an easement = 0.246 + (0.086 *house) + (0.060 *road) + (-0.020 *slope) + (0.003 *water) + (0.123 *diversity) + (0.268 *elevation) + (1.157 *income) + (-0.862 *trust) + (0.158 *nccpi) + (0.151 *urban) + (-0.317 *protect) + (-0.190 *income *income) + (-0.121 *road *road) + (-0.137 *urban *urban) + (-0.079 *nccpi *nccpi) + (0.105 *diversity *diversity) + (0.255 * urban *elevation) + (0.327 *income *trust) + (0.287 *income *density) + (-0.261 *elevation *density) + (0.171 *urban *density) + (-0.201 *income *protect) + (0.158 *road *diversity) + (-0.176 *urban *protect) + (-0.174* slope *density) + (0.128* density *diversity) + (0.124 *income *slope) + (-0.140 *diversity *protect) + (-0.132 *road *density) + (0.109* slope *diversity) + (-0.122 *trust *protect) + (-0.099 *urban *nccpi) + (0.088 *water *slope) + (0.067 *water *nccpi). See Table 1 for full variable names.
